# Risks, benefits size and clinical implications of combined oral contraceptive use in women with polycystic ovary syndrome

**DOI:** 10.1186/s12958-017-0313-y

**Published:** 2017-12-08

**Authors:** Sebastião Freitas de Medeiros

**Affiliations:** 1 Department of Gynecology and Obstetrics, Medical School, Federal University of Mato Grosso, Cuiabá, MT 78055-728 Brazil; 2 Tropical Institute of Reproductive Medicine, Cuiabá, MT Brazil

**Keywords:** Polycystic ovary syndrome, Contraception, Oral contraceptive, Progestin, Venous thromboembolism, Dyslipidemia, Dysglicemia

## Abstract

**Background:**

Polycystic ovary syndrome (PCOS) is a complex condition with high risk for dyslipidemia, dysglycemia, venous thromboembolism, cardiovascular disease and metabolic syndrome. Because the combined oral contraceptive (COC) use has also been associated with impaired fasting glucose, insulin resistance and increased risk of thromboembolism disease, it is rationale to think that the combination of oral contraceptive and PCOS could make it worse or increase the risks.

**Objective:**

To examine the current data regarding potential additional risks and benefits of contraceptive use, highlights the major gap in knowledge for designing future studies and, when possible, suggests an adequate COC formulation for a determined PCOS phenotype.

**Methods:**

English-language publications reporting on the influence of COCS in the development of venous thromboembolism in PCOS patients published until 2017 were searched using PubMed, Cochrane database, and hand search of references found in consulted articles. Ranges of collected data are given; the pooled data are presented as median and first and third quartiles. Wilcoxon signed-ranks test for paired samples was used to compare before-after original data. *P* value was set at 0.05.

**Results:**

Most of COCs preparations significantly decrease androgens, and increase sex-hormone binding globulin. Therefore, the benefits of COCs are clear in patients with proved hyperandrogenemia. Regarding the impact of COCs on carbohydrate metabolism of PCOS subjects, the data were inconsistent but they tended to show no additional risk. Regarding lipids, most COCs consistently increased high-density lipoprotein cholesterol, triglycerides and total cholesterol concentrations but the clinical implications of these changes need additional studies.

**Conclusion:**

The review showed consistent beneficial effect of COCs, particularly for hyperandrogenemic PCOS patients. The benefit size of COC’s use by normoandrogenemic PCOS patients is uncertain and need more investigation. The effects of COC use on carbohydrate metabolism of women with PCOS are still unresolved since most studies are observational but the current results demonstrated that COCs do not make their levels worse and may improve insulin sensitivity. The impact of COCs on lipids of PCOS patients seems to be clearer and most preparations increase total cholesterol, high-density lipoprotein cholesterol and triglycerides. In summary, it is important to balance the potential benefits and risks of the COCs individually before prescribing them for PCOS women.

## Background

The prevalence of polycystic ovary syndrome (PCOS) ranges from 5% to 22% in women of reproductive age worldwide. It is associated with menstrual disturbances, hyperandrogenism, infertility, obesity and insulin resistance [1, 2]. Dyslipidemia, dysglycemia and metabolic syndrome (MetS) are also common [2, 3]. Adding evidences suggest that non-alcoholic fatty liver disease (NAFLD) exacerbates insulin resistance and predisposes patients with PCOS to an atherogenic status and the release of many proinflammatory, procoagulant and profibrogenic moderators [4]. Collectively, these data demonstrate that PCOS patients exhibit many conditions that could be tightly linked to future development of cardiovascular disease (CVD) and venous thromboembolism (VTE) (Table 1).

**Table 1:** Abnormal and subclinical conditions associated with risk of venous thromboembolism and cardiovascular disease in polycystic ovary syndrome

Central obesity	
Systemic arterial hypertension	
Hyperandrogenemia	
Impaired glucose tolerance	
Insulin resistance	
Type II diabetes mellitus	
Metabolic syndrome	
Dyslipidemia	
Nonalcoholic fatty liver disorder	
Low-grade chronic inflammatory disease	
Obstructive sleep apnea	
Subclinical vascular disease	

Despite the complex pathophysiological mechanisms and multiple phenotypes of PCOS, combined oral contraceptives (COCs) are the first-line option for the treatment of all women with PCOS not seeking to become pregnant, and they exert many beneficial effects in these women [5, 6]. However, some deleterious metabolic effects have been demonstrated [7]. Concerns regarding the safety and the potential metabolic and cardiovascular risks of COCs in these patients persist [7–11]. This review has several purposes: (a) to consider the potential risks of isolated PCOS; (b) to estimate the potential additional risks of COC in patients with PCOS; (c) to examine why the use of COCs would be either beneficial or harmful for PCOS patients; (d) to focus on the non-contraceptive advantages/disadvantages of COC use in this syndrome; and (e), when possible, to suggest a specific formulation according to the patients’ phenotype.

## Methods

A comprehensive search and review of published studies on this topic and written in the English-language between 1982 and 2017 was performed using PubMed and the Cochrane database. When available, online searches of specialized journals were also used by adding adequate keywords. In addition, these databases were expanded by a manual search of references from the obtained articles. Older articles were also included as they provided basic and essential information. Keywords, alone or in combination, for the search included oral contraceptive, progestin, polycystic ovary syndrome, venous thromboembolism, cardiovascular disease and contraception. On the basis of the literature search, eligible studies that met the inclusion criteria of comparing head-to-head before and after data were identified. Ranges of collected data are given and pooled data are presented as median and first and third quartiles. Wilcoxon signed-ranks test for paired samples was used to compare before-after original data. *P* value was set at 0.05.

## Impact of combined oral contraceptives in the risk of VTE and CVD in PCOS patients

An isolated diagnosis of PCOS increases the risk for VTE and CVD when compared with the general population [12–14]. VTE is considered the third most common cardiovascular disease with an overall annual incidence estimated at 1–2 events for every 1000 adults per year [15], has multiple risk factors and PCOS is a possible predisposing condition [13]. The likelihood of VTE among women with PCOS is reported to be 1.5–3 times higher than in non-PCOS controls [13, 16–19] and the risk can be modified by age, PCOS phenotype, oral contraceptive use, liraglutide [14] and comorbidities such as central obesity, diabetes mellitus type 2 (T2DM), or (MetS) [13]. Table 2 shows the size of potential factors of risk of VTE and CVD in PCOS non-users of COCs when compared with non-PCOS subjects [12, 13, 18, 20–25]. Regarding PCOS phenotype, the influence of higher androgen levels on the risk of VTE remains controversial [12, 13, 19, 23, 26, 27].

**Table 2:** Risks of venous/arterial diseases in PCOS women when compared with non-PCOS controls

Variable	Risk (95%CI)^a^	Reference
VTE	1.55 (1.10–2.19)	ST Bird et al., 2013
	3.26 (2.60–4.08)	EM Okoroh et al., 2012
CHD	1.50 (0.70–2.90)	S Wild et al., 2000
	1.25 (1.07–1.47)	CG Solomon et al., 2002
	1.63 (0.96–2.78)	SA Anderson et al., 2014
	1.27 (1.10–1.46)	EM Okoroh et al., 2012
	1.44 (1.13–1.84)	L Zhao et al., 2016
Stroke	2.80 (1.10–7.10)	S Wild et al., 2000
	1.30 (0.97–1.74)	CG Solomon et al., 2002
	1.61 (0.94–3.315)	SA Anderson et al., 2014
	2.20 (1.50–3.20)	T Matthesen et al., 2015
CHD/Stroke	2.02 (1.47–2.76)	PCM de Groot et al., 2011
	4.24 (1.96–9.17)	D Cibula et al., 2000

*VTE* Venous thromboembolism, *CHD* Coronary heart disease, *CI* 95% confidence interval

^a^Relative risk or hazard risk or odds ratio

Since a diagnosis of PCOS alone might increase the risk of VTE and CVD, it is important to consider the possible existence of additional risk factors for these patients, including the use of COCs [10, 11, 28]. Simple disturbances in sex-steroid secretion, which are commonly seen in PCOS, has been associated with an increased risk of atherosclerosis and adverse cardiovascular events [18]. The proper use of COCs has also been associated with a two-fold increased risk of fatal and nonfatal cardiovascular events [28, 29], which are perhaps related to endothelial dysfunction by altering the levels of nitric oxide, homocysteine, and angiotensin [30]. It must be emphasized that the association between the use of COCs and CVD has been demonstrated even in non-PCOS subjects, mainly among women who concomitantly smoke, who are over 35 years old or are users of third- or fourth-generation COCs that contain less androgenic progestin [31–35].

Three studies have examined the risk of VTE in users of COCs with PCOS (Table 3). One study using a combination of 35 μg ethinylestradiol (EE) and 2 mg cyproterone acetate (CPA) detected an odds ratio of 2.2 (95% CI, 1.35–3.58) and 7.44 (95% CI, 3.67–15.08) for the risk of VTE compared with users of other COCs and to non-users, respectively [36]. One cross-sectional study included women who were 18–45 years old and who used any kind of COC demonstrated a protective association (OR = 0.8; 95% CI, 0.73–0.98) in PCOS users [13]. On the other hand, a Canadian study that included women who were 18–46 years old and used different criteria to diagnosis PCOS and different COCs containing <35 μg EE combined with second and third-generation progestins found a two fold increased risk for VTE in PCOS users (RR = 2.14, 95% CI, 1.41–3.24), with a risk that was even higher in women with hyperandrogenism (HR = 2.49; 95% CI, 1.35–4.59) [18]. In summary, the results of these studies have limitations because they did not use the same criteria for the diagnosis of PCOS, they compared subjects using a different combination of COCs and did not control for confounding variables such as PCOS phenotype, diet, exercise or body mass index.

**Table 3:** Comparison of venous thromboembolism risks in PCOS users versus PCOS non-users of combined oral contraceptive

Variable	Risk (95% CI)^c^	Reference
VTE	2.58 (1.60–4.18)^a^	HE Seaman et al., 2003
	7.44 (3.67–15.08)^b^	HE Seaman et al., 2003
	0.80 (0.73–0.98)	EM Okoroh et al., 2012
	2.14 (1.41–3.24	ST Bird et al., 2013

*CI* 95% confidence interval, *VTE* Venous thromboembolism

^a^Any combined oral contraceptive

^b^Combined oral contraceptive containing ethynilestradiol and cyproterone acetate

^c^Relative risk

## Potential benefits of combined oral contraceptives in PCOS

Although, women with PCOS commonly present with hyperandrogenemia, central obesity, dysglycemia, dyslipidemia, and MetS, COCs are the preferred treatment intervention for women who do not wish to become pregnant. The reasons to choose COCs are sustained by several pharmacological benefits: a decrease in LH pulsatile secretion, a reduction in total and free circulating testosterone, an inhibition of 5 α-reductase enzyme activity, an increase in sex-hormone binding globulin (SHBG) levels, diminishing free testosterone levels (fT), an increase in insulin muscle sensitivity, menses regulation, elimination of the clinical signs of hyperandrogenism, and protection of the endometrium against neoplasms [3, 10]. An ideal COC combination for PCOS women have not been established [6, 37], although COC use is currently considered to be safe [6, 24, 38, 39]. Those patients with important metabolic risk factors should have an individualized approach [10, 40, 41]. The impact of COCs on the metabolic markers of PCOS was recently reviewed [10, 42].

### Sex-hormone binding globulin

SHBG, glycoprotein produced by hepatocytes in the liver, binds to testosterone and estradiol with a high affinity. Its synthesis is increased by estrogen, thyroxin and decreased by androgens. Progesterone and progestins have variable androgenic effects and counter the beneficial estrogen effect on SHBG according to the androgenicity of each progestin. Low plasma SHBG levels which are seen in PCOS patients are considered an independent risk factor for the development of CVD [43–46], and NAFLD [47]. A few mechanisms may be involved in this association [48] and to increase SHBG concentrations in PCOS patients is necessary [49]. Since the relative androgenicity of progestin can be assessed by the measurement of plasma SHBG, this procedure may be useful in the clinical practice for assessing the estrogen/androgen balance of COCs. The estrogen components of COCs (e.g., ethinylestradiol and estradiol) induce hepatic SHBG production in a different intensity [50, 51].

Concentrations of SHBG using a combined formulation that contains 35 μg EE and 2 mg of cyproterone acetate (CPA) for 3–6 months was reported in twelve studies [52–63]. The size of the SHBG increase found with this combination ranged from 37.31 nmol/l to 179.01 nmol/l, with an average increase of 141.7 nmol/l (*p* = 0.002). It has been accepted that the combination induces the highest rise in SHBG and that is the most estrogenic COC in relation to its higher contents of estrogen and to the low androgenicity of CPA [56, 64]. Therefore, if the primary outcome is to increase SHBG, it was shown that 2 mg CPA had no counteractive effect on the increase of SHBG levels induced by EE. Therefore this formulation is recommended, mainly for the treatment of PCOS patients with proved biochemical hyperandrogenism and menstrual disturbances.

Fifteen studies have reported on the SHBG increase with a COC combination containing of 30 μg ethynilestradiol (EE) and 3 mg drospirenone (DRSP) for six months. [52, 54, 62, 65–76]. The increase in SHBG concentrations with this combination ranged from 35.8 mmol/l to 140.8 mmol/l, to an average increase of 105 mmol/l (*p* < 0.001). Recently, two studies [76, 77] using 3 mg DRSP with 20 μg EE demonstrated an increase in SHBG levels from 40.0 mmol/l to 125.1 mmol/l, an increment of 85 nmol/l (*p* = 0.074). Only a single recent double-blind controlled trial reported a nonsignificant decrease of 0.5 mmol/l in the SHBG level with a pill that contained DRSP (*p* > 0.05) [78]. Therefore, regarding SHBG concentrations the benefits with the use of 20 μg EE associated with 3 mg DRSP are very low for clinical recommendation.

The impact of 2 mg of chlormadinone acetate (CMA) combined with 30 μg EE on SHBG concentrations was verified in six studies [67, 73, 75, 79–81]. SHBG levels increased from 38.8 mmol/l to 181.9 mmol/l, average of 143.1 mmol/l (*p* = 0.027). As SHBG secretion is not counteracted by CMA, which is bound to albumin rather than to SHBG, the current review support the knowledge that the combination is adequate for PCOS patients, primerly to those hyperandrogenemic with very low SHBG levels [82]. With a combination of 30 μg EE and 150 μg desogestrel (DSG) in PCOS patients, SHBG blood levels were examined in five studies [52, 73, 74, 83, 84]. An increase in SHBG concentrations between 30 nmol/l and 123 nmol/l was detected, with an average of +46.9 nmol/l (*p* = 0.043). A biphasic COC containing 40 μg EE for the first 7 days plus 25 μg DSG and 30 μg EE plus 125 μg DSG for the subsequent 14 days increased SHBG from 43.2 nmol/l to 177.2 nmol/l, increase of 134 nmol/l (*p* < 0.01) [55]. This combined COC containing DSG blocks the estrogen-mediated increase in SHBG more than other antiandrogenic progestins [74] and would not be the first choice for women with PCOS, mainly for those with biochemical hyperandrogenism.

A few studies reported on the use of COCs containing 30 μg EE with 75 μg gestodene (GSD) in PCOS patients. The results of two open clinical studies [73, 85] reported an increase in SHBG levels from 21.7 nmol/l to 109.2 nmol/l, average increase of 65.4 nmol/l (*p* = 0.049). Because the few number of studies and the high affinity of GSD for SHBG, no conclusion regarding the increase of SHBG could be done. Only one study [86] reported on SHBG levels modification in PCOS women using a combination of EE 35 μg/NGM 250 μg and, in this study, SHBG changed from 32 nmol/l to 180 nmol/l, an increase of 148 nmol/l (*p* < 0.001) after 6 months of use. One other study evaluated the impact of EE30 μg/LNG 150 μg on SHBG and demonstrated an increase from 126 nmol/l to 195 nmol/l, absolute increase of 69 nmol/l (p < 0.001) [86]. When EE 20 μg was combined to LNG 150 μg, the increase in SHBG varied from 57.7 nmol/l to 201 nmol/l, absolute change of 143 nmol/l (*p* < 0.05) [87]. A synthesis of the impact of different COCs on SHBG levels is shown in Table 4.

**Table 4:** Influence of different combined oral contraceptive formulations in the hyperandrogenemic biomarkers of polycystic ovary syndrome

COC formulation	Number of studies	Before Md (1st – 3rd)	After Md (1st – 3rd)	∆	p*
SHBG (nmol/l)					
EE 35 μg / CPA 2 mg	12	37.3 (32.7–52.9)	179.0 (122.1–235.7)	+141.7	0.002
EE 30 μg / DRSP 3 mg	15	35.8 (29.9–45.3)	140.8 (62.8–180.0)	+105.0	<0.001
EE 30 μg / CMA 2 mg	06	38.8 (27.0–42.9)	181.9 (140.0–217.9)	+143.1	0.027
EE 30 μg / DSG 75–150 μg	05	30.0 (22.5–30.0)	76.0 (60–129)	+46.0	0.043
EE 30 μg / GSD 75 μg	02	21.7 (17.5–26.0)	109.2 (87–131.0)	+65.4	0.049
Total T (ng/dl)					
EE 35 μg / CPA 2 mg	11	79.5 (31.2–100.0)	46.9 (30.0–66.1)	−32.5	0.003
EE 30 μg / DRSP 3 mg	15	66.5 (60.0–86.4)	48.9 (39.0–55.0)	−17.6	<0.001
EE 30 μg / CMA 2 mg	05	78.0 (73.4–84.1)	41.9 (45.8–60.3)	−36.1	0.043
EE 30 μg / DSG 75–150 μg	07	52.3 (44.0–67.4)	36.1 (31.0–42.0)	−16.2	0.027
EE 30 μg / GSD 75 μg	02	72.7 (64.4–81.0)	51.8 (47.5–56.0)	19.9	0.179
Free T (pmol/l)					
EE 35 μg / CPA 2 mg	07	4.3 (2.8–8.8)	2.4 (1.5–5.9)	−1.9	0.011
EE 30 μg / DRSP 3 mg	05	2.2 (2.2–2.4)	1.9 (1.1–1.96)	−0.3	0.043
EE 30 μg / CMA 2 mg	05	7.8 (6.1–7.9)	4.2 (3.8–4.7)	−3.6	0.043
EE 30 μg / DSG 150 μg	04	2.6 (1.6–6.8)	1.4 (1.0–1.7)	−1.2	0.067
FAI (%)					
EE 35 μg / CPA 2 mg	07	10.2 (5.4–11.5)	2.1 (0.8–2.8)	−8.0	0.042
EE 30 μg / DRSP 3 mg	14	6.8 (5.1–9.3)	1.2 (0.9–4.2)	−5.6	0.001
EE 30 μg / CMA 2 mg	05	9.4 (7.9–9.7)	0.9 (0.9–0.94)	−8.5	0.043
EE 30 μg / DSG 75–150 μg	05	4.8 (3.0–6.4)	0.7 (0.6–0.74)	−4.1	0.043

*COC* combined oral contraceptive, *Md (1st -3rd)* median, first and third quartiles, *EE* ethinylestradiol, *CPA* cyproterone acetate, *DRSP* drospirenone, *CMA* chlormadinone acetate, *DSG* desogestrel, *GSD* gestodene, *SHBG* sex-hormone binding globulin, *T* testosterone, *FAI* free androgen index

p* Wilcoxon signed- ranks test

### Total testosterone

Testosterone (T) is the most potent circulating androgen in women [50]. Approximately 65–70% of circulated T is bound and inactivated by SHBG, 30–35% is bound by albumin, and 1–3% represents the free T fraction [88]. The decrease of total and free testosterone is a primary objective for the use of COCs in PCOS patients. The benefit of COCs in hyperandrogenic PCOS patients is determined by two mechanisms: increasing SHBG for binding the T and decreasing the blood concentrations of the active free T molecule [50, 89]. Regarding this matter, both the dose of the estrogen component and the androgenicity of the progestin must be considered. A summary of all studies published in this field is depicted in Table 4.

A combination of 35 μg EE plus 2 mg CPA in PCOS was reported on T concentrations at baseline and after six months of use in eleven studies [53, 55–60, 62, 63, 65, 90]. In all these studies, T concentrations showed a significant decrease from 79.53 ng/dl to 46.97 ng/dl, an average decrease of 32.56 ng/dl (*p* = 0.003). It can be concluded that in PCOS patients a decrease of about 30 ng/dl in the T levels would be expected with this combination. The results confirm the knowledge that the preparation is suitable for hyperandrogenemic PCOS women.

Using a combination of 30 μg EE and 3 mg DRSP, the decrease in T levels in PCOS patients was reviewed in fifteen studies [52, 62, 65–71, 73–77, 91]. In two publications, the extent of the modification of the T levels was not accurately verified [52, 75]. Considering all thirteen studies in which was possible to calculate T changes, this formulation decreased T levels from 66.53 ng/dl to 48.9 ng/dl, with an average decrease of 17.6 ng/dl (*p* < 0.001). It seems that DRSP, even presenting a more prominent adrenal influence [54], significantly decrease total testosterone levels of PCOS users and could be chosen when patients present hypertension or hyperandrogenism.

The impact of a 30 μg EE with 2 mg CMA on T concentrations in PCOS patients was reported in five studies [67, 73, 75, 79, 81]. The significant decrease of T ranged from 78 ng/dl to 41.9 ng/dl, with an average decrease of 36.1 ng/dl (*p* = 0.043). Collectively, these results indicate that the combination can significantly decrease total testosterone levels in PCOS patients and could be a good option for PCOS patients with the hyperandrogenemic phenotype.

Seven studies reported changes in T concentrations in PCOS patients using a combination of 30 μg EE and 75 μg DSG: [52, 55, 73, 83, 85, 92, 93]. With this preparation the decrease in T levels ranged from 52.3 ng/dl to 36.1 ng/dl, with an average decrease of 16.21 (*p* = 0.027). In summary, it can be considered the use of COCs containing DSG in PCOS patients is a reasonable second option when a decrease in T levels is the main objective. Only two open clinical trials reported a combination of 30 μg EE with 75 μg GSD in PCOS patients [73, 85]. In these reports, T levels decreased from 72.7 ng/dl to 51.8 nmol/l, average decrease of 19.9 nmol/l (*p* = 0.179). Because GSD competes with testosterone for SHBG sites, COCs containing this progestin should be avoided in PCOS patients, or at least considered a poor alternative.

### Free testosterone

Approximately 1–3% of testosterone circulates as free T (fT). High fT in PCOS women are linked to low levels of SHBG and high levels of testosterone secretion [89]. The imprecision of the assays commonly used for fT measurement in women in the various studies limits some of the conclusions. Twenty one studies that reported on the impact that COCs had on the fT concentrations of PCOS patients were reviewed (Table 4). Seven of these studies have examined what the combination 35 μg EE with 2 mg CPA had on the fT levels [53, 55, 56, 83, 90, 94, 95]. They have shown a decrease in free testosterone concentrations that ranged from 4.31 pg/ml to 2.4 pg/ml, with an average decrease of 1.9 pg/ml (*p* = 0.011).

The impact that 30 μg EE combined with 3 mg DRSP had on fT concentrations was reported on five open clinical trials [54, 68, 71, 73, 96] which reported that fT levels decreased from 2.2 pg/ml to 1.9 pg/ml, with an average decrease of 0.3 pg/ml (*p* = 0.043). Patients included in the study by Batukan and Muderris presented hirsutism but had not had a diagnosis of PCOS confirmed [71]. Different combinations of 30–40 μg EE with 25–125 mg DSG were reported to have affected the fT levels in PCOS patients [55, 73, 89]. With these combinations the decrease in fT levels ranged from 2.6 pg/ml to 1.4 pg/ml, with an average of −1.2 pg/ml (*p* = 0.067). In five open trial [67, 73, 75, 79, 81] and a case-control study [67] a combination of 30 μg EE with 2 mg CMA decreased fT from 7.8 pg/l to 4.2 pg/ml in PCOS patients, with an average decrease of 3.6 pg/l (*p* = 0.043).

The combination of 30 μg EE and 150 μg DSG, evaluated in four studies [55, 73, 83, 92], decreased fT from 2.6 pg/ml to 1.4 pg/ml, an average decrease of 1.2 pg/ml (p = 0.067), demonstrating that the preparation is not an adequate choice for treating PCOS patients. A combination of 30 μg EE with 2 mg DNG decreased fT from 1.4 pg/ml to 0.56 pg/ml (*p* < 0.01) [50, 97] and when 2 mg DNG was combined with 2 mg valerate estradiol (VE), fT decreased from 3.6 to 3.0 pg/ml (16%) after six months and from 3.6 to 2.5 ng/ml (22%) after twelve months (*p* > 0.05) [98], reflecting the lower impact of VE on the liver secretion of SHBG when compared with ethinylestradiol. Other COCs combining 20 μg. EE with 100 μg levonorgestrel (LNG) and 30 μg EE with 75 μg GSD decreased fT levels from 1.56 pg/ml to 0.63 pg/ml from (*p* < 0.01) and 2.0 pg/ml to 1.2 pg/ml (*p* < 0.05) [50, 73], respectively. Only one study compared the effect of the combination of EE 35 μg/NGM 180–250 μg in the fT levels in PCOS patients, fT decreased from 0.81 pg/ml to 0.35 pg/ml (*p* < 0.05) [99].

### Free androgen index

The FAI decrease in hyperandrogenemic women has been considered an independent predictor of improvement in endothelial function in these patients [59, 100]. Thirty one studies, reported on the decrease of the FAI in PCOS patients (Table 4). The combination of 35 μg EE with 2 mg CPA was examined in seven studies [52, 54, 58–60, 62, 63]. Taking the results of these studies as a whole group, the FAI decreased from 10.2% to 2.1%, with an average decrease of 8% (*p* = 0.042). Using the combination of 30 μg EE and 3 mg of DRSP changes in the FAI levels were reported in fourteen studies [52, 54, 62, 65–67, 69, 70, 72, 74–77, 101]. With this combination, FAI decreased from 6.8% to 1.2%, with an average decrease of 5.6% (*p* = 0.001). This combination may be the preferred choice for PCOS patients with hyperandrogenemia, particularly whether associated with arterial hypertension or obesity. Prospective clinical trials remain needed though.

The effect of 2 mg CMA and 30 μg EE on FAI in PCOS patients was verified in five studies [67, 73, 75, 80, 81], and a significant decrease in FAI ranged from 9.4% to 0.92%, with an average decrease of 8.5% (*p* = 0.043). Collectively, these studies indicate that CMA does not counteract the liver’s EE effect on the secretion of SHBG, assure a remarkable decrease in the FAI, and demonstrate that it is an adequate option for the treatment of hyperandrogenemic PCOS patients. In PCOS patients, a significant decrease in the FAI level was also reported in five studies with the COC combination of 25–150 μg DSG and 30–40 μg EE [55, 61, 74, 92, 93]. The FAI decreased from 4.82% to 0.7%, with an average decrease of 4.1% (p = 0.043). Only one open clinical trial reported on the decrease of the FAI in PCOS patients using the COC combination of 75 μg GSD and 30 μg EE [85]. The FAI decreased from 13.3% to 2.2% (*p* < 0.05), after twelve months of follow-up; this study did not report on FAI decrease within six months, as was reported by the other studies analysed in this review. It may be concluded that at the moment this combination should not be a first-line choice for PCOS patients. Taken all studies united, it may be concluded that formulations containing CPA, CMA, and DRSP diminish the FAI by 80% - 85% and should be preferred in PCOS patients.

### High-density lipoprotein-cholesterol

The impact of COCs containing 35 μg EE and 2 mg CPA on the HDL-C levels in PCOS patients was reported in ten studies [53, 56, 59, 61, 63, 90, 94, 95, 100, 102]. Taking all of them, HDL-C levels significantly increased from 54.3 mg/dl to 61.5 mg/dl, with an average increase of 7.3 mg/dl (*p* = 0.020). Only in three of these studies, HDL-C presented a nonsignificant increase [53, 59, 94]. In a single study HDL-C did not change in PCOS patients who used this formulation [95]. COCs with DRSP significantly increased the HDL-C levels of PCOS patients from 59.5 mg/dl to 73.3 mg/dl, average of 13.8 mg/dl (*p* = 0.017) [54, 69, 72, 74, 91, 96, 100, 101]. In two particular studies, the HDL-C nonsignificantly increased by 5% [54, 96] in DRSP preparations.

The influence of COCs containing 30 μg EE and 2 mg CMA on HDL-C concentrations in PCOS patients was examined in three studies only [75, 80, 81]. HDL-C increased from 51.1 mg/dl to 61.4 mg/dl, average of 10.3 mg/dl (*p* = 0.108). Three other studies [61, 74, 103] reported an decrease in the HDL-C levels from 48.6 mg/dl to 45.3 mg/dl, average of 3.3 mg/dl (*p* = 0.592) in PCOS patients who used COCs containing DSG. COCs with GSD [85] presented a nonsignificant decrease in the HDK-C, from 46.7 mg/dl to 45.9 mg/dl (*p* > 0.05). Collectively, the results indicated that the increase in HDL-C with most COC preparations has clinical implications and should be considered a beneficial effect.

## Potential harms of combined oral contraceptives in polycystic ovary syndrome

Considering the different phenotypes, many PCOS patients may present with a slight increase in blood pressure, be overweight or obese, and present with dysglycemia, dyslipidemia, MetS, NAFLD and elevated concentrations of chronic inflammatory process mediators. The use of different COC preparations may cause some harmful effects. Further, the phenotypes of women with PCOS should be additionally considered, and the decision to choose a specialized COC formulation should be made based on the estrogen type and dose and the progestin androgenicity [50, 104]. Potential risks or disadvantages of COC use in PCOS women are considered according to the available data.

### Blood pressure

Considering all formulations, fourteen studies reported on the influence of COCs on the systolic blood pressure (SBP) of PCOS patients [52, 59, 72, 74, 75, 77, 80, 81, 99–103, 105]. Four studies reporting on 35 μg EE with 2 mg CPA use demonstrated a nonsignificant increase in the SBP, from 114.2 mmHg to 116.19 mmHg, average increase of 2 mmHg (*p* = 0.144) [52, 59, 77, 103] of women with PCOS. With the combination of 30 μg EE and 3 mg DRSP, seven studies [52, 72, 74, 75, 77, 100, 101] reported a nonsignificant decrease in SBP, from 118.9 mmHg to 118.4 mmHg, average decrease of 0.5 mmHg (*p* = 0.730). Only the Battaglia study demonstrated a significant rise in the SBP [72]. SBP diminished from 113.9 mmHg to 112.2 mmHg, average decrease of 1.7 mmHg (*p* = 0.108) with the use of a COC preparation containing 30 μg EE and 2 mg CMA [75, 80, 81]. Only one single study showed a nonsignificant increase in SBP in PCOS patients that used this preparation [80]. Oral contraceptives containing 30 μg EE and 75–150 μg DSG was reported to increase SBP from 107.3 mmHg to 108.2 mmHg, difference of 0.9 mmHg (*p* = 0.179) [74, 105]. Two recent publications reporting on the use of COC containing NGM did not find any modification in SBP [99, 105]. In summary, regarding SBP, the available data indicate that there are inconsistent results and nonsignificant changes in baseline SBP with the use of COCs, should not be expected, independent of the formulation. The possible beneficial effect of COC combination containing DRSP in the SBP was not proved in this review and this matter must be considered in future well-designed clinical trials.

The impact of 2 mg CPA combined with 35 μg EE on the diastolic blood pressure (DBP) was reported in five studies [52, 59, 77, 85, 103] and demonstrated a nonsignificant increase in DBP from 75.9 mmHg to 78.8 mmHg, an increase of 2.8 mmHg (*P* = 0.067). Therefore, choosing this formulation for PCOS patients with arterial hypertension might not be recommended. Seven studies examined the effect of COCs containing 30 μg EE and 3 mg DRSP on DBP [52, 72, 74, 75, 77, 100, 101] and demonstrated a nonsignificant increase from 75 mmHg to 75.7 mmHg, with an increment of 0.7 mmHg (*p* = 0.398). Interesting to note that two of these studies reported nonsignificant decreases of 0.5% [74] and 7.6% [75] in the DBPs of PCOS patients with this combination. Nonsignificant decrease in DBP, from 74.4 mmHg to 71.5 mmHg, decrease of −2.9 mmHg (*p* = 0.102) was demonstrated with the use of COCs containing 2 mg CMA in PCOS patients [75, 80, 81]. Two studies evaluated the impact of COCs containing 75–150 μg DSG on the DBP of PCOS patients and found a nonsignificant increase from 69.0 mmHg to 70.5 mmHg, increment of 1.5 mmHg (*p* = 0.500) [74, 103]. Collectively, it could be concluded that COCs, independently of the combination, do not significantly influence the DBPs of PCOS patients who use them. However, pills with CMA might be preferred in PCOS patients with arterial hypertension.

### Total body weight

The analysis of four RCTs revealed no difference in body weight gain after six months of COC use in non-PCOS women [106]. In addition, an observational study following COC users for more than two decades also did not demonstrate significant weight gain [107]. Eleven studies have examined 3 to 6 months impact of COCs on the body weight (BW) of PCOS patients [59, 63, 67, 69, 74, 79, 81, 84, 92, 96, 102]. Regarding the use of EE combined with CPA, two studies reported a nonsignificant increase from 69.6 kg to 69.8 kg, increase of 0.2 kg (*p* = 0.754). Therefore, this formulation does not influence the BW of PCOS users.

The use of COC containing 30 μg EE with 3 mg DRSP resulted in a nonsignificant decrease in BW from 63.5 kg to 63.0 kg, average decrease of 0.5 kg (*p* = 0.465) [67, 69, 74, 96]. The use of COCs containing 30 μg EE and 2 mg CMA in PCOS patients was reported in three studies [75, 80, 81] that resulted in a nonsignificant BW increase the range from 57.7 kg to 58.1 kg, average increase of 0.4 kg (*p* = 0.653). PCOS patients who used 30 μg EE combined with 150 μg DSG had a nonsignificant BW increase from 67 kg to 68.2 kg, increase of 1.2 kg (*p* = 0.900) [74, 92]. In summary, most of the different COC formulations did not promote significant weight gain in PCOS patients (Table 5). However, there is no strong evidence from the current data, it has been suggested that COC formulations with DRSP might be the right choice for obese PCOS women.

**Table 5:** Influence of different combined oral contraceptive formulations in the anthropometric parameters of polycystic ovary syndrome

COC formulation	Number of studies	Before Md (1st – 3rd)	After Md (1st – 3rd)	∆	p*
BW					
EE 35 μg / CPA 2 mg	02	69.6 (67.7–80.2)	69.8 (67.1–72.3)	+0.2	0.754
EE 30 μg / DRSP 3 mg	04	63.5 (60.1–66.2)	63.0 (61.8–65.1)	−0.5	0.465
EE 30 μg / CMA 2 mg	03	57.5 (55.9–59.7)	58.1 (56.7–61.3)	+0.4	0.653
EE 30 μg / DSG 75–150 μg	02	67.0 (66.1–67.9)	68.2 (67.5–70.1)	+1.2	0.900
BMI (kg/m^2^)					
EE 35 μg / CPA 2 mg	13	24.0 (22.6–27.3)	23.1 (22.1–26.9)	−0.9	0.151
EE 30 μg / DRSP 3 mg	17	23.4 (22.2–26.4)	24.5 (22.7–26.3)	+1.1	0.924
EE 30 μg / CMA 2 mg	05	22.5 (22.2–23.3)	22.7 (22.5–23.4)	+0.2	0.138
EE 30 μg / DSG 75–150 μg	07	26.7 (24.4–30.1)	27.2 (23.8–29.2)	+0.5	0.262
WC (cm)					
EE 30 μg / DRSP 3 mg	03	76.7 (74.0–79.4)	77.6 (75.4–79.2)	+0.9	0.810
EE 30 μg / CMA 2 μg	02	78.3 (78.0–78.7)	72.2 (77.0–77.4)	−1.1	0.089
EE 30 μg / NGM 180–250 μg	03	80.3 (63.3–97.3)	79.7 (63.7–95.7)	−0.6	0.179
WHR					
EE 35 μg / CPA 2 mg	07	0.80 (0.74–0.91)	0.79 (0.74–0.88)	−0.01	0.179
EE 30 μg / DRSP 3 mg	12	0.78 (0.76–0.81)	0.75 (0.74–0.81)	−0.03	0.241
EE 30 μg / CMA 2 mg	02	0.73 (0.51–0.095)	0.78 (0.75–0.81)	−0.05	0.317
EE 30 μg / DSG 75–150 μg	04	0.81 (0.75–0.82)	0.80 (0.75–0.82)	−0.01	0.654
EE 30 μg / NGM 180–250 μg	02	0.80 (0.74–0.86)	0.78 (0.75–0.81)	−0.02	0.317
FM (kg)					
EE 30 μg / DRSP 3 mg	03	21.0 (19.20–22.0)	22.9 (20.9–24.8)	+1.9	0.108

*COC* combined oral contraceptive, *Md (1st – 3rd)* median, first and third quartiles, *BW* body weight, *BMI* body mass index, *WC* waist circumference, *WHR* waist-hip ratio, *FM* fat mass, *EE* ethinylestradiol, *CPA* cyproterone acetate, *DRSP* drospirenone, *CMA* chlormadinone acetate, *DSG* desogestrel, *NGM* norgestimate

p* Wilcoxon signed- ranks test

### Body mass index

Thirteen studies evaluated the impact of COCs containing 35 μg EE and 2 mg CPA on the body mass index (BMI) of PCOS patients [52–55, 58–60, 62, 63, 94, 95, 100, 102]. Four studies demonstrated a significant decrease in the BMI of PCOS patients [94, 95, 100, 102], while other four studies showed a nonsignificant decrease in the BMI [52, 54, 55, 58]. Only six studies reported a nonsignificant increase in the BMI of PCOS patients who used CPA [53, 59, 60, 62, 63, 67]. Collectively, these studies demonstrated a slight decrease in the BMI of PCOS patients who use EE combined with CPA, from 24 kg/m^2^, to 23.1 kg/m^2^ a decrease of 0.9 kg/m^2^ (*p* = 0.151). BMI changes in PCOS users of COCs containing 30 μg EE and 3 mg DRSP were reported on seventeen clinical studies [52, 54, 62, 65–69, 72, 74–77, 91, 96, 100, 101]. Two studies reported a significant BMI decrease in PCOS patients [74, 100] and nine studies found a nonsignificant decrease [52, 54, 65, 66, 69, 72, 75, 79, 96]. Only six studies reported a nonsignificant increase in the BMI of PCOS patients who used COCs containing DRSP [62, 67, 69, 76, 77, 101]. Collectively, these studies demonstrated a nonsignificant small increase in the BMI from 23.4 kg/m^2^ to 24.47 kg/m^2^, an increase of 1.02 kg/m^2^(*p* = 0.924) with the use of COC with DRSP.

BMI changes in PCOS patients using a combination of EE with CMA was reported in five studies [67, 75, 79–81]. Four of the studies reported a nonsignificant BMI increase [67, 79–81]. Only the study by Yildizhan found a nonsignificant decrease of 0.5% [75]. Taken together, with this combination the BMI increased from 22.52 kg/m^2^ to 22.7 kg/m^2^, an increase of 0.18 kg/m^2^ (*p* = 0.138). Therefore, this COC does not modify the BMI of PCOS patients who use it. DSG combined with EE was shown to decrease BMI in a nonsignificant range in seven studies [52, 55, 74, 92, 103, 105, 108]. Either nonsignificant or significant increases of 1.1% and 5.3% in the BMIs of PCOS patients was also shown in two studies [74, 103]. Seven studies with EE combined with DSG demonstrated that BMI increased from 26.7 kg/m^2^ to 27.2 kg/m^2^, an increase of 0.5 kg/m^2^ (*p* = 0.262). GSD associated with EE induced a nonsignificant increase in the BMI from 26.2 kg/m^2^ to 26.5 kg/m^2^ in a single study (*p* > 0.05) [85]. Recently, it was shown that COC with NGM decreased the BMI from 32.6 kg/m^2^ to 32.5 kg/m^2^ (p > 0.05) [99]. In conclusion, the current studies did not endorse the idea that COCs increase BMI (Table 6).

**Table 6:** Influence of different combined oral contraceptive formulations in the carbohydrate metabolism biomarkers of polycystic ovary syndrome

COC formulation	Number of studies	Before Md (1st – 3rd)	After Md (1st – 3rd)	∆	p*
Glucose (mg/dl)					
EE 35 μg / CPA 2 mg	12	86.6 (81.0–92.0)	85.6 (84.3–91.9)	−1.0	0.514
EE 30 μg / DRSP 3 mg	10	84.4 (78.7–91.2)	85.7 (80.6–90.6)	+1.3	0.998
EE 30 μg / CMA 2 μg	2	86.0(84.5–87.5)	81.8 (80.5–83.1)	−4.2	0.010
EE 30 μg / DSG 75–150 μg	5	84.5 (81.3) – 88.9)	85.2 (81.9–88.0)	+0.7	0.892
Insulin (μUI/ml)					
EE 35 μg / CPA 2 mg	11	17.9 (10.8–22.8)	17.1 (10.0–18.6)	−0.8	0.213
EE 30 μg / DRSP 3 mg	08	13.9 (11.3–18.1)	12.1 (10.1–18.5)	−1.8	0.865
EE 30 μg / CMA 2 mg	03	7.3 (5.2–9.4)	7.8 (6.1–9.5)	+0.5	0.285
EE 30 μg / DSG 75–150 μg	04	15.7 (13.0–19.0)	17.1 (13.1–22.1)	+1.4	0.144
EE 30 μg / 180–250 μg NGM	02	8.9 (7.6–10.2)	9.1(7.2–11.0)	+0.2	0.788
C-peptide (mg/dl)					
EE 35 μg / CPA 2 mg	03	2.6 (2.0–3.2)	3.0 (2.9–3.9)	+0.4	0.179
EE 30 μg / DRSP 3 mg	03	1.2 (0.7–1.7)	1.3 (0.7–1.9)	+0.1	0.654
EE 30 μg / DSG 75–150 μg	02	1.6 (1.5–1.7)	2.4 (2.0–2.8)	+0.8	0.179
HOMA-IR					
EE 35 μg / CPA 2 mg	09	3.6 (2.0–4.4)	2.2 (1.8–3.9)	−1.4	0.213
EE 30 μg / DRSP 3 mg	10	3.1 (2.2–3.7)	2.8 (2.5–3.8)	−0.3	0.593
EE 30 μg / CMA 2 mg	03	1.6 (1.2–2.0)	1.6 (1.3–2.0)	0	0.592
EE 30 μg / DSG 75–150 μg	03	3.0 (2.3–3.7)	2.6 (2.2–3.0)	−0.4	0.285
HbA1C (%)					
EE 35 μg / CPA 2 mg	04	5.0 (4.6–5.4)	5.1 (4.7–5.6)	+0.1	0.422
EE 30 μg / DRSP 3 mg	02	3.5 (2.7–4.3)	3.5 (1.7–7.3)	0	0.653

*COC* combined oral contraceptive, *Md (1st – 3rd)* median, first and third quartiles, *HOMA-IR* homeostatic model assessment for insulin resistance, *HbA1C* glycated hemoglobin, *EE* ethinylestradiol, *CPA* cyproterone acetate, *DRSP* drospirenone, *CMA* chlormadinone acetate, *DSG* desogestrel, *NGM* norgestimate

p* Wilcoxon signed- ranks test

### Waist circumference

Possible waist circumference (WC) changes with the use of COCs in PCOS patients were examined in nine studies [52, 59, 69, 77, 80, 81, 93, 99, 109]. One study reported a nonsignificant increase of 0.1 cm (76.3 cm to 76.4 cm) in the WC [54], when a COC formulation containing CPA was used. A combination of EE with DRSP also showed nonsignificant increases from 76.7 cm to 77.6 cm, increment of 0.9 cm (*p* = 0.810) in the WC of PCOS patients, [52, 69, 77]. COCs containing EE and CMA showed a nonsignificant decrease in the WC in PCOS users, which was from 78.3 to 77.1 cm, decrease of 1.1 cm (*p* = 0.179) [80, 81]. The combination of 30 μg EE plus 180–250 μg NGM showed to diminish WC from 80.3 cm to 79.7 cm (p = 0.179) [93, 99, 109]. The analysis of nine studies reporting on the influence of different COCs in the WC allowed to conclude that COCs do not change significantly this anthropometric parameter. This conclusion confirms the findings of a recent systematic review [107].

### Waist-hip ratio

Seven studies examined the influence of 35 μg EE with 2 mg CPA on the waist-hip ratio (WHR) of PCOS patients [52–55, 60, 100, 102]. Five studies showed no modification of the WHR with this combination, and two studies showed a nonsignificant decrease in the WHR [53, 102]. The WHR changed from 0.8 cm to 0.79 cm (p = 0.179) when all studies were considered for calculation. Therefore, COCs with CPA had no impact on the WHR of PCOS patients and can be used in patients with an abnormal WHR. The influence of COCs containing 30 μg EE and 3 mg DRSP in the WHR of PCOS patients were examined in twelve studies [52, 54, 65, 66, 69, 70, 72, 75–77, 100, 101]. Nonsignificant decrease from 0.78 cm to 0.75 cm was found, average decrease of 0.03 cm (*p* = 0.241). It can also be concluded that COCs containing DRSP has little to no effect on the WHR of PCOS patients.

The influence of COCs containing 30 μg EE and 2 mg CMA on the WHR of PCOS patients were examined in only two studies [75, 79] that demonstrated an increase from 0.73 to 0.78 in this ratio (*p* = 0.317). The impact of 30 μg EE combined with 75–150 μg DSG was reported in four clinical studies [52, 55, 92, 93] and the WHR decreased from 0.81 to 0.80 (*p* = 0.654); therefore, it can be concluded that COCs containing either CMA or DSG also have no impact on the WHR of PCOS users. Formulation containing 30 μg EE and 180–250 μg NGM showed a nonsignificant decrease in WHR, from 0.80 to 0.78 (*p* = 0.317) in two studies [99, 109]. Collectively, COCs use by PCOS patients do not modify the WHR (Table 5).

### Fat mass

The influence of COC use in the fat mass (FM) of PCOS women have been examined in seven studies [69, 79, 91, 96, 102, 106, 110]. FM (kg) was reported to increase in PCOS patients who used 30 μg EE with 3 mg DRSP in three studies [69, 91, 96]. This increase was from 21 kg to 22.9 kg, increase of 1.9 kg (*p* = 0.108). A nonsignificant decrease of 0.16% was also reported in a single study [110]. Changes in FM were not reported in PCOS patients who used other COC formulations [79, 102]. The percentage of body fat mass (FM%) exhibited a nonsignificant decrease of 0.4% in a single study [102]. A significant decrease of 6.5% was found in another study [69], but the FM % was significantly increased by 9% in an earlier publication [96]. Currently, it is not possible to make any conclusive statement regarding the effect of COCs on the body fat change in PCOS patients, although long-term COC use tends to reduce abdominal fat depots [107].

### Carbohydrate metabolism

The effect of COCs on the carbohydrate metabolism of non-PCOS subjects has been associated with increased fasting glucose, increased fasting insulin and abnormal oral glucose tolerance test (OGTT). However, the results are inconsistent. This section examines the impact of different COC combinations on the carbohydrate metabolism of PCOS users. A summary of the current data is provided in Table 6.

#### Fasting glucose

Fasting glucose, in PCOS patients who used 35 μg EE with 2 mg CPA, was described in twelve studies published between 1990 and 2016. Had a nonsignificant increase in fasting glucose levels ranged from 0.57% to 2.57%, average of 1.2%, demonstrated in seven studies [56, 58–60, 90, 102, 105]. Three clinical trials reported no change in fasting glucose with this preparation [52, 54, 63], and a nonsignificant decrease of 4.0% [55] and a significant decrease of 8.0% [53] were also detected. As a whole group, the studies demonstrated that fasting glucose levels decreased from 86.6 mg/dl to 85.5 mg/dl (*p* = 0.514) in PCOS users of COC with CPA. A recent study examining a three-hour OGTT response found a nonsignificant decrease from 460.6 to 450.8 (*p* = 0.359) in the glucose area under the curve (AUC) [105]. Collectively, these findings indicate that there is a nonsignificant increase, or even a slight decrease, in the fasting glucose levels of PCOS patients who used COCs containing CPA.

With the use of COCs containing DRSP, the fasting glucose levels of PCOS patients presented nonsignificant increase in four clinical studies [69, 72, 96, 110]. In another three studies, the fasting glucose decreased in a nonsignificant way [52, 66, 101]. However, fasting glucose levels decreased significantly in two other reports [74, 100], but in one single study, fasting glucose levels did not change with COCs containing DRSP [54]. Collectively, these ten studies showed a nonsignificant increase of fasting glucose from 84.4 mg/dl to 85.7 mg/dl (*p* = 0.998). In addition, three studies reported the glucose AUC after OGTT in PCOS patients who used COCs with DRSP. Two of these studies showed nonsignificant increases of 0.4% [72] and 9% [110]; the other study demonstrated a significant increase from 419.8 to 467.8 (*p* = 0.005) [100]. At this moment, it is not possible to make conclusive statement regarding the impact of COCs containing DRSP on the glucose metabolism of PCOS users, but the current studies indicate that there is no additional harm.

Fasting glucose changes with the use of COCs containing CMA in PCOS patients were reported to decrease it from 86 mg/dl to 81.8 mg/dl, decrease of 4.2 mg/dl (*p* = 0.010) [80, 81]. In five studies [52, 55, 74, 92, 103] the use of COC preparations with DSG showed a nonsignificant increase in fasting glucose ranging from 84.5 mg/dl to 85.2 mg/dl, an increase of 0.7 mg/dl (*p* = 0.892). In conclusion, the use of COCs with DSG does not significantly modify the fasting glucose levels of PCOS patients. The combination of EE 33 μg and NGM 180–250 μg in PCOS women showed a nonsignificant decrease in the fasting glucose from 86.2 mg/dl to 85.1 mg/dl (*p* = 0.179) [99, 109, 111]. In summary, COCs containing CMA, DSG, and NGM also do not modify the fasting glucose levels in PCOS users.

#### Fasting insulin

Eleven prospective clinical studies have reported on the impact of COCs containing CPA in the fasting insulin levels of PCOS patients. Four studies reported a nonsignificant decrease [53, 59, 60, 102], but two reported significant decreases [94, 95]. Nonsignificant increases were also reported with the use of CPA [52, 55, 63, 100]. A single study did not demonstrate any modification in the fasting insulin of PCOS patients who used COCs containing CPA [90]. Regarding fasting insulin levels, collectively, these studies demonstrated that insulin decreased from 17.9 μUI/ml to 17.1 μUI/ml (*p* = 0.213). Therefore, two studies reported significant decreases in the insulin AUC after OGTT in PCOS users of CPA [59, 100]. Additionally, these studies strongly indicated that COCs containing CPA do not worsen insulin metabolism in PCOS patients.

The impact of 30 μg EE with 3 mg DRSP on the fasting insulin of PCOS patients was reported in eight studies. Six studies reported nonsignificant increases [52, 54, 69, 72, 78, 96], one single study reported an astonishing increase of 241% in the fasting insulin levels with the use of DRSP, but this study had a very small sample size and a very low power [96], and another study reported a nonsignificant decrease [101]. Significant decreases in fasting insulin were also demonstrated with EE plus DRSP [78, 100]. In addition, eight studies have shown that fasting insulin decreased from 13.9 μUI/ml to 12.1 μUI/ml, a decrease of 1.8 μUI/ml (*p* = 0.865). One study also reported a very small and clinically nonsignificant decrease of the insulin AUC after OGTT [72]. However, a clinically significant decrease of 20.3% in the insulin AUC was recently reported [100]. On the other hand, a nonsignificant increase of 31% in the AUC was also found [96]. In summary, COCs with DRSP can be used safely in PCOS patients and do not appear to cause any additional disturb in the fasting insulin.

The use of COCs containing 30 μg EE with 2 mg CMA has shown inconsistent results regarding fasting insulin levels. Both, a nonsignificant decrease [79] and a nonsignificant increase [80, 81] have been reported. Considering all three studies, the insulin levels increased from 7.3 μUI/ml to 7.8 μUI/ml (*p* = 0.285). COCs containing 30 μg EE and 75–150 DSG increased the insulin levels of PCOS patients from 15.7 μUI/ml to 17.1 μUI/ml, an increase of 1.4 μUI/ml (*p* = 0.144) [52, 55, 74, 92]. It can be concluded that COCs containing DSG tend to increase the fasting insulin levels of PCOS users. The use of 30 μg EE and 180–250 μg NGM increased insulin levels from 8.9μUI/ml to 9.1μUI/ml (*p* = 0.788) in two studies [99, 111]. Collectively, COCs with CMA, DSG, and NGM progestins promote a small and nonsignificant increase in the fasting insulin levels of PCOS patients.

#### C-peptide

The influence of COCs containing 35 μg EE and 2 mg CPA in the C-peptide concentrations of PCOS patients have been reported in a few studies, and the results are inconsistent. A significant increase in C-peptide of 28.9% was previously reported using this preparation [60]. Either a nonsignificant increase [55] or no change [54] in C-peptide levels have also been found. Taking these three studies together, C-peptide levels increased 0.4 ng/ml, from 2.6 ng/ml to 3.0 ng/ml (*p* = 0.179) in PCOS patients with this combination. The effect of COCs with DRSP in the C-peptide levels of PCOS patients are also unclear: a nonsignificant decrease of 4.7% [72] and nonsignificant increases of 3% [54] and 10% [110] were reported. In fact, COCs with DRSP increased C-peptide from 1.2 ng/ml to 1.3 ng/ml in these three studies (*p* = 0.654). The AUC of the C-peptide was shown to increase by a nonsignificant 11.6% in PCOS patients who used COCs with DRSP [110].

A formulation containing CMA with EE showed a nonsignificant decrease in the C-peptide levels from 4.43 ng/ml to 4.35 ng/ml (*p* = 0.317) [75] and COC with DSG presented an nonsignificant increase of 0.7 ng/ml, from 1.63 ng/ml to 2.38 ng/ml (p = 0.179) [55, 93]. Thus, the results of these studies do not allow for any conclusive statement regarding the effect of all COC preparations on the C-peptide concentrations of PCOS patients. The impact of these preparations on the C-peptide concentrations must be specifically re-examined.

#### Homeostatic model assessment-IR

HOMA-IR changes in PCOS patients who used COCs containing CPA were reported in nine prospective studies. A nonsignificant decrease in the HOMA-IR index, was demonstrated in five of those studies [54, 59, 62, 100, 103], a significant decrease in two [94, 95] and a nonsignificant increases in other two studies and CPA [52, 63]. Collectively, HOMA-IR decreased from 3.6 to 2.2 (*p* = 0.213) in PCOS users of COCs with this formulation. In summary, regarding HOMA-IR, COCs with CPA can be safely used in PCOS patients and might improve insulin resistance.

With the combination of 30 μg EE and 3 mg DRSP changes in the HOMA-IR of PCOS patients wer reported in ten studies. Five of then showed a nonsignificant increase [52, 54, 63, 66, 69]; one single study showed a significant increase, and another one [72] demonstrated a significant decrease of 31% [100]. Two studies reported a nonsignificant decrease in the HOMA-IR with DRSP pill [74, 75] and, in addition, no modification of the HOMA-IR was shown in a recent double-blind RCT [101]. Collectively, all ten studies demonstrated that HOMA-IR had a nonsignificant small decrease from 3.1 to 2.8 (*p* = 0.593) with the use of DRSP preparations. Therefore, the data regarding the impact of a pill with DRSP on the HOMA-IR are inconsistent, but not harmful.

The HOMA-IR was shown to increase from 1.58 to 1.63 (*p* = 0.592) in PCOS patients who used COCs containing 30 μg EE and 3 mg CMA [75, 80, 81]. One single study showed a nonsignificant decrease in this index with this preparation [75]. COCs containing DSG showed either an increase [74] or a decrease [52, 108] in the HOMA-IR of PCOS patients. The analysis of these three studies demonstrated that HOMA-IR decreased from 3.0 to 2.6 (*p* = 0.285) with pill containing DSG. In summary, COCs containing most progestins present inconsistent effects in the HOMA-IR of PCOS patients and more studies are required to take a definitive conclusion.

#### Glycated haemoglobin

Glycated hemoglobin (HbA1C) may be a better indicator of overall glycemia status in both non-PCOS [112] and PCOS individuals [113]. HbA1C levels have been reported to be increased in PCOS patients when compared with non-PCOS controls [114–116]. Some guidelines suggest that the measurement of HbA1C may be an acceptable option to screen for dysglycemia [5, 117] and to discriminate patients in whom OGTT would be appropriated [2]. Currently, changes in the HbA1C levels of PCOS patients using COCs were reported in a few studies. Two studies did not show any modification in HbA1C levels with EE plus CPA preparation [54, 100]. Otherwise, two prospective trials showed a nonsignificant increase in the HbA1C levels of PCOS patients who used this preparation [58, 61]. Mutually, these studies have demonstrated a nonsignificant increase in HbA1C, from 5.0 to 5.1 (*p* = 0.422) with CPA pill HbA1C levels maintained unchanged with the use of COCs containing EE 30 μg and DRSP 3 mg: from 3.5 to 3.5 after six months (*p* = 0.653). Therefore, no definitive conclusion can be drawn at this time regarding the impact of COCs containing CPA or DRSP on the HbA1C in PCOS patients who used these preparations for six months.

### Lipid metabolism

As dyslipidemia is common in PCOS women, it is recommended that lipid measurement is performed before any therapeutic intervention. The impact of different COC preparations on the lipid levels of non-PCOS subjects have been extensively investigated. This section describes the main discoveries of observational and randomized and non-randomized controlled trials currently available for analysis regarding the effect of different COC combinations on the lipids of PCOS patients. Table 7 illustrates a summary of the current discoveries.

**Table 7:** Influence of different combined oral contraceptive formulations in the lipid concentrations of polycystic ovary syndrome

COC formulation	Number of studies	Before Md (1st – 3rd)	After Md (1st – 3rd)	∆	p*
Total Cholesterol (mg/dl)					
EE 35 μg / CPA 2 mg	12	173.7 (162.1–184.1)	189.0 (185.3–200.9)	+15.3	0.012
EE 30 μg / DRSP 3 mg	09	171.5 (166.8–185.9)	185.2 (167.3–203.1)	13.7	0.085
EE 30 μg / DSG 75–150 μg	03	158.9 (154.9–162.9)	178.8 (169.0–188.4)	+19.5	0.108
EE 30 μg / 180–250 μg NGM	02	168.9 (167.6–170.2)	181.7 (181.3–182.1)	+12.8	0.060
EE 30 μg / 150 μg LNG	02	161.8 (158.4–165.2)	170.4 (169.1–173.8)	+8.6	0.265
LDL-C (mg/dl)					
EE 35 μg / CPA 2 mg	11	98.0 (91.0–103.9)	101.9 (86.8–114.6)	+3.9	0.241
EE 30 μg / DRSP 3 mg	08	95.2 (87.6–107.0)	101.3 (92.6–113.1)	+6.1	0.345
EE 30 μg / CMA 2 μg	03	101.5 (99.6–103.4)	100.9 (98.3–103.5)	+0.6	0.486
EE 30 μg / DSG 75–150 μg	03	94.3 (94.0–94.5)	106.8 (100.9–112.7)	+12.5	0.108
EE 30 μg / NGM 180–250 μg	02	100.2 (99.9–100.5)	107.6 (106.7–108.3)	+7.4	0.031
EE 30 μg / LNG 150 μg	02	87.2 (77.4–97.0)	90.6 (80.2–101.0)	+3.4	0.022
HDL-C (mg/dl)					
EE 35 μg / CPA 2 mg	10	54.3 (50.2–59.5)	61.5 (60.7–67.2)	+7.3	0.020
EE 30 μg / DRSP 3 mg	08	59.5 (50.0–62.5)	73.3 (64.5–79.7)	+13.8	0.017
EE 30 μg / CMA 2 mg	03	51.1 (49.9–52.3)	61.4 (61.1–61.7)	+10.3	0.108
EE 30 μg / DSG 75/150 μg	03	49.6 (34.7–59.7)	45.3 (26.0–75.0)	−3.3	0.592
TG (mg/dl)					
EE 35 μg / CPA 2 mg	11	81.4 (69.0–99.1)	104.0 (85.8–123.4)	+22.6	0.021
EE 30 μg / DRSP 3 mg	07	95.0 (72.8–105.6)	116.8 (115.0–138.9)	+21.8	0.017
EE 30 μg / CMA 2 mg	03	83.9 (83.6–84.2)	125.5 (116.1–134.9)	+41.6	0.108
EE 30 μg / DSG 75–150 μg	03	87.7 (83.7–91.7)	108.3 (102.4–114.2)	+20.6	0.137
EE 30 μg / NGM 180–250 μg	03	104.7 (86.1–123.3)	134.3 (117.4–151.3)	+29.6	0.025

*COC* combined oral contraceptive, *Md (1st – 3rd)* median, first and third quartiles, *LDL-C* low-density lipoprotein cholesterol, *HDL-C* high-density lipoprotein cholesterol, *TG* triglyceride, *CPA* cyproterone acetate, *DRSP* drospirenone, *CMA* chlormadinona acetate, *DSG* desogestrel, *NGM* norgestimate, *LNG* levonorgestrel

p* Wilcoxon signed- ranks test

#### Total cholesterol

The influence of COCs containing 35 μg EE with 2 mg CPA on the total cholesterol (TC) concentrations of PCOS patients have been investigated in twelve studies and 2016. Five of them reported nonsignificant increases [54, 56, 58, 90, 102]. Additionally, five other studies reported significant increases in the TC levels of PCOS patients [53, 59, 61, 63, 100]. One single study reported a nonsignificant decrease [95] and another study reported a significant decrease in TC levels [94]. The analysis of these twelve studies as a whole group, demonstrated that TC levels increased from 173.7 mg/dl to 189.1 mg/dl, an increase of 15.4 mg/dl (*p* = 0.012). Overall, it can be concluded that the use of COC containing CPA by PCOS patients causes a significant increase in TC concentrations. So, its use in dyslipidemic PCOS patients should not be recommended.

The role of COCs with DRSP on the TC levels of PCOS patients were examined in nine studies. Two studies reported a nonsignificant increase of TC [70, 96], six studies demonstrated a significant increase [54, 69, 72, 74, 75, 100], and one single study demonstrated a significant decrease [101]. In combination, all nine studies presented a nonsignificant increase in the TC levels, from 171.5 mg/dl to 185.2, an increment of 13.7 mg/dl (*p* = 0.085). As a whole group, the studies demonstrated that COCs with DRSP also increase TC levels in PCOS patients, of course with small and low intensity than COCs with CPA in their formulation.

In one single study EE combined with CMA showed a significant increase in the TC levels, increasing it from 161.1 mg/dl to 193.2 mg/dl (*p* < 0.05) [81]. The impact of COCs with 30 μg EE and 75–150 μg DSG on the TC of PCOS patients were an increase from 158.9 mg/dl to 178.8 mg/dl, increase of 20 mg/dl (*p* = 0.108) [61, 74, 103]. A preparation with GSD increased the TC levels from 188.4 mg/dl to 198.8 mg/dl (*p* > 0.05) [85]. Combined 30 μg EE and 180–250 μg NGM increased TC levels from 168.9 mg/dl to 181.7 mg/dl (*p* = 0.060) in two studies [99, 111]. COCs with LNG increased TC from 161.8 mg/dl to 170.4 mg/dl (*p* = 0.265) [86, 103]. Collectively, the results indicate that most COC preparations increase the TC levels of PCOS patients and, currently, there does not exist a preferred formulation. This fact must be considered for proper COC prescription in dyslipemic conditions.

#### Low-density lipoprotein cholesterol

The possible changes in LDL-C levels in PCOS patients who used COCs containing CPA was examined in eleven clinical studies. Three studies presented nonsignificant decreases in LDL-C [56, 90, 102] and five other studies have found nonsignificant increases in the LDL-C [53, 54, 59, 95, 103]. Two studies demonstrated a significant increase [61, 63] and another one showed a significant decrease [100] in the LDL-C levels with the CPA pill. As a whole, these studies demonstrated that with the pill containing CPA, LDL-C increased from 98 mg/dl to 101.9 mg/dl (*p* = 0.241). Therefore, the results regarding LDL-C levels in PCOS patients who used COCs containing CPA are inconsistent but leaning towards a small nonsignificant increase.

PCOS patients using COCs with DRSP may have a nonsignificant decrease in their LDL-C levels [91, 96]. In addition, a significant decrease was recently found [74, 101]. Besides, two studies have reported significant increases in LDL-C levels [69, 100] and two other studies also have found nonsignificant increases [54, 72]. Therefore, data regarding LDL-C levels in PCOS patients who used a pill with DRSP are inconsistent. Collectively, COCs with this combination increased LDL-C levels from 95.2 mg/dl to 101.3 mg/dl (*p* = 0.345) and these results allowed to concluded that regarding LDL-C levels, COCs with DRSP have no additional risk in PCOS patients.

The influence of COCs with CMA in the LDL-C levels of PCOS patients are insignificant [75, 80, 81] and LDL-C levels changed from 101.5 mg/dl to 100.9 mg/dl (*p* = 0.486) with this preparation. COCs containing DSG induced a nonsignificant increase from 94.3 mg/dl to 106.8 mg/dl (*p* = 0.108) [61, 74, 103] in the LDL-C levels of PCOS patients. One single study reported a nonsignificant increase from 112.7 mg/dl to 117.7 mg/dl (*p* = 0.053) in the LDL-C levels of PCOS patients who used COCs containing GSD [85]. COCs with EE 30 μg and NGM 180–250 μg increased LDL-C from 100.2 mg/dl to 107.6 mg/dl (*p* = 0.031) [99, 111]. The combination of 30 μg EE and 150 μg LNG increased LDL-C from 87.2 mg/dl to 90.6 mg/dl (*p* = 0.022) [86, 103]. Collectively, the studies reporting the impact of COCs on the LDL-C levels are inconsistent but combinations with GSD, LNG, and NGM progestins might be preferred in opposite to those with CPA and DRSP progestins.

#### Triglycerides

The impact of COC preparations containing CPA on the TG levels were reported in eleven clinical studies. It was demonstrated a significant increase of TG levels in seven studies [56, 59–61, 63, 90, 102]. Nonsignificant decrease in two other studies were also described [53, 94]. In one single study, the TG levels did not change at all [98], and in another trial, a significant decrease was found with this preparation [100]. The analysis of all eleven studies using the Wilcoxon test showed that TG levels were increased from 81.4 mg/dl to 104 mg/dl (*p* = 0.021). Therefore, COCs containing the CPA progestin significantly increase TG levels of PCOS patients.

EE combined with DRSP that was used by PCOS patients produced a significant increase in TG concentrations in six studies [69, 72, 74, 91, 96, 100] and this increase was not significant in just one study [75]. Testing the seven studies as a whole group, TG levels increased from 95 mg/dl to 116.8 mg/dl, a difference of 21.8 mg/dl (*p* = 0.017). So, COCs containing DRSP significantly increase TG levels in blood. COCs with CMA also increased the TG levels of PCOS patients from 83.9 mg/dl to 125.5 mg/dl, a nonsignificant increase of 41.6 mg/dl (*p* = 0.137) [75, 80, 81]. DSG combined with 30 μg EE produced a nonsignificant TG level increase from 87.7 mg/dl to 108.3 mg/dl (*p* = 0.108) [74, 103, 117].

The pill with GSD also increased LDL-C from 112.7 mg/dl to 117.7 mg/dl (*p* > 0.05) [85]. The combination of 30 μg EE with NGM significantly increased TG levels from 104.7 mg/dl to 134.3 mg/dl (*p* = 0.025) [99, 109, 111]. COCs combining EE and LNG increased TG levels from 136.1 mg/dl to 102.5 mg/dl (*p* = 0.377) [86, 103]. Collectively, it can be considered that COCs, independent of progestin preparation, can increase the TG levels of PCOS patients. Therefore, the presence of hypertriglyceridemia should be considered before any COC prescription. Combinations containing CMA, and DSG seem to present a lower impact on TG levels of PCOS women.

## Conclusions

Collectively, the available studies, either randomized controlled trials or prospective observational, suggest that the use of different COCs preparations do not get worse or bring additional harm for PCOS users, at least when used for 3–6 months. Because of remarkable decrease in androgen and increase in SHBG concentrations, hyperandrogenemic PCOS phenotype patients may have greater benefits and antiandrogenic progestins are preferred in patients with this phenotype. PCOS patients with hypertension, or central obesity, can use any COC preparation, however evidence is lacking. Scarce data suggests that preparations containing antiandrogenic/antimineralocorticoid progestin could be chosen. Regarding carbohydrate metabolism changes in PCOS patients, the published data indicate that COCs do not make it worse or significantly increase baseline blood concentrations of them. Apart from that, COCs with DRSP and DSG may decrease HOMA-IR improving tissue insulin sensitivity. TC, TG, and HDL-C are consistently increased by COCs but the clinical implications of these changes still need more long-term investigation. Despite the fact that there is no clear advantage of one preparation over other, it is recommended that lipid baseline levels should be measured to help chosing a particular COC preparation for dyslipidemic patients.
